# Oxidative Stress in Cardiovascular Diseases and Obesity: Role of p66Shc and Protein Kinase C

**DOI:** 10.1155/2013/564961

**Published:** 2013-03-27

**Authors:** Elena De Marchi, Federica Baldassari, Angela Bononi, Mariusz R. Wieckowski, Paolo Pinton

**Affiliations:** ^1^Department of Morphology, Surgery and Experimental Medicine, Section of General Pathology, Interdisciplinary Center for the Study of Inflammation (ICSI), Laboratory for Technologies of Advanced Therapies (LTTA), University of Ferrara, Via Borsari 46, 44121 Ferrara, Italy; ^2^Department of Biochemistry, Nencki Institute of Experimental Biology, 3 Pasteur, 02093 Warsaw, Poland

## Abstract

Reactive oxygen species (ROS) are a byproduct of the normal metabolism of oxygen and have important roles in cell signalling and homeostasis. An imbalance between ROS production and the cellular antioxidant defence system leads to oxidative stress. Environmental factors and genetic interactions play key roles in oxidative stress mediated pathologies. In this paper, we focus on cardiovascular diseases and obesity, disorders strongly related to each other; in which oxidative stress plays a fundamental role. We provide evidence of the key role played by p66^Shc^ protein and protein kinase C (PKC) in these pathologies by their intracellular regulation of redox balance and oxidative stress levels. Additionally, we discuss possible therapeutic strategies aimed at attenuating the oxidative damage in these diseases.

## 1. Introduction

Obesity, high blood pressure, insulin resistance, and aging are associated with the development of cardiovascular diseases (CVDs), and all these factors are correlated with metabolic syndrome (MS) [1]. Lifestyle, environmental, genetic, and epigenetic interactions reflect complex pathological processes [2] in which the oxidative stress caused by reactive oxygen species (ROS) plays a pivotal role. ROS are not only considered to be the damaging factors in various pathologies, but they also participate in a wide variety of physiological processes such as insulin-signalling transduction [3, 4]. Mitochondria are the primary source of ROS production and the major target for their damaging effects [5]. Therefore, mitochondrial ROS production and oxidative damage may contribute to the onset and progression of these pathologies. CVDs, obesity, diabetes, and atherosclerosis are also the result of interactions between excessive weight and lifestyle, environmental, and genetic factors. 

This paper aims to illustrate the correlation between oxidative stress, obesity, and CVDs, especially focusing on the 66-kilodalton (kDa) isoform of the growth factor adapter Shc (p66^Shc^) and some isoforms of the protein kinase C (PKC) family that are particularly sensitive to redox stress and are implicated both in CVDs and obesity [6–8].

## 2. Generation of ROS and Oxidative Stress: An Overview

ROS generation can be finely controlled and can constitute a physiologic signalling pathway. The enzyme systems responsible for ROS generation, as well as the antioxidant defences, have specific subcellular localization and thus give rise to the concept of compartmentalization of both ROS production and the signalling response. ROS can originate from different subcellular sources, but mitochondria are generally considered the primary source of ROS generation [9, 10]. ROS are produced at a low level by the electron transport chain as a normal part of oxidative phosphorylation and play a physiologically important role in the regulation of cell signalling, proliferation, and differentiation. However, oxidative phosphorylation also generates ROS, since a proportion of O_2_ molecules (1–3%) taken up by cells are converted into superoxide anion radicals (O_2_^•−^) by complexes I and III. This radical can subsequently be diverted into hydrogen peroxide (H_2_O_2_) and the hydroxyl radical (OH^•^). Such oxidative species are considered normal metabolic by-products. They are continuously generated by mitochondria and are kept in check by endogenous cellular antioxidant mechanisms, such as superoxide dismutase (which rapidly converts superoxide into H_2_O_2_ and O_2_), catalase, glutathione peroxidase, and peroxiredoxins distributed throughout the cell.

Oxidative stress represents an imbalance between ROS production and the cellular antioxidant defence system. In stress conditions, ROS levels increase and, because of their high reactivity, participate in a variety of chemical reactions. They are involved in cell damage, necrosis, and apoptosis via oxidation of lipids, proteins, and DNA [11] and provoke also endothelial dysfunction, infiltration, and activation of inflammatory cells [12]. 

ROS production can rise when the breakdown of metabolites in the tricarboxylic acid (TCA) cycle exceeds the capacity of the electron transport chain (ETC) to assimilate the resulting electrons [13]. While O_2_^•−^ mediates its effects within a short range of its production, H_2_O_2_ is more stable and can diffuse throughout the cell; hence, despite the compartmentalization of ROS production, electrons generated by excess mitochondrial metabolism can be used to regulate intracellular signalling through the production of ROS [10]. Moreover, ROS can be transferred across cell membranes through several mechanisms. H_2_O_2_ can diffuse through aquaporin channels in the plasma membrane to elicit an intracellular signalling response. Aquaporins belong to a large family of proteins that form pores in the membrane and conduct water in and out the cell [14], and H_2_O_2_ has almost the same size, dielelectric properties, and capacity to form hydrogen bonds as does water. Bienert et al. provided molecular genetic evidence that aquaporins, and in particular hAQP8, AtTIP1;1, and AtTIP1;2, can channel H_2_O_2_ [15]. Also extracellular O_2_^•−^ can initiate intracellular signalling by penetration of the cell membrane through anion channels (chloride channel-3, ClC-3) [16].

## 3. ROS in Cardiovascular Diseases: Role of p66^Shc^ and PKC

CVDs are a class of pathologies involving the heart or blood vessels (arteries, capillaries, and veins). They refer to any disease that affects the cardiovascular system, mainly cardiac diseases, vascular diseases of the brain and kidney, and peripheral arterial disease. World Health Organization (WHO) data published in September 2012 define CVDs as the principal cause of death globally: more people die annually from CVDs than from any other cause.

### 3.1. ROS Implications in Cardiovascular Diseases

Oxidative stress has a central role in the pathogenesis of atherosclerosis; indeed, it is a critical feature in atherogenesis. An increased generation of ROS in the vascular wall and a reduction of nitric oxide (NO) bioavailability lead to endothelial dysfunction in atherogenesis [17, 18]. ROS cause damage to cellular structures within the vascular wall, and they trigger several redox-sensitive transcriptional pathways, shifting the cell towards a proatherogenic transcriptomic profile. Animal models of atherosclerosis demonstrate the involvement of ROS in atherosclerosis by the accumulation of lipid peroxidation products and induction of inflammatory genes [19] and activation of matrix metalloproteinases [20]. ROS and reactive nitrogen species (RNS) produced by the endothelium promote oxidative modification of LDL (low-density lipoprotein) in the phase that precedes the transfer into the subendothelial space of the arterial wall, where they initiate atherosclerosis [21]. 

An important source of ROS is represented by NADPH oxidases (Nox), a family of enzyme complexes that catalyze the transfer of electrons from NADPH to molecular oxygen to generate O_2_^•−^. Important roles have been shown for NADPH oxidases in redox signalling events involved in hypertension, atherosclerosis, endothelial activation, and angiogenesis, as well as in endothelial dysfunction [22]. The close functional association between NADPH oxidase and the renin-angiotensin system may be of particular relevance in linking oxidative stress to hypertension [23]. The excess generation of ROS contributes to the development of CVDs, particularly atherosclerosis. NADPH oxidase is in fact present in the macrophage [24], and O_2_^•−^ inactivates NO promoting endothelial activation [25]. Nox2 and Nox4 are the most abundant NADPH oxidases in the heart and are expressed in cardiomyocytes, endothelial cells, and fibroblasts [26]. Interestingly, a recent study by Judkins et al. shows that in apolipoprotein E-null (ApoE^−/−^) mice maintained on a high-fat diet, Nox2 deletion was associated with decreased aortic ROS production and markedly less atherosclerotic plaque formation [27]. Recent studies by Shimizu et al. also confirmed the contribution of Nox1-derived ROS in modification of lesion composition and atherosclerosis [28]. Moreover, Nox4, a member of the NADPH oxidases (Nox) family expressed primarily in mitochondria in cardiac myocytes, was reported to be a major source of superoxide production in the cardiovascular system. Nox4 mediates cardiac hypertrophy and heart failure in response to pressure overload. Upregulation of Nox4 increased mitochondrial superoxide thereby directly mediating oxidative stress, mitochondrial dysfunction, and myocardial cell death during pressure overload-induced cardiac hypertrophy [29]. Some of the most compelling evidence that mitochondrial ROS are causative agents in the development of CVDs *in vivo* comes from experiments using transgenic mice to alter expression of mitochondrial antioxidant proteins. Initial experiments using genetic knockouts showed that mice lacking MnSOD produce huge amounts of mitochondrial ROS and develop cardiomyopathy within the first weeks of birth [30]. Nowadays, it is widely accepted that deficiencies in mitochondrial antioxidants and/or regulatory proteins that modulate mitochondrial oxidant production promote the onset of CVDs.

Smoking, hypertension, and diabetes mellitus, which represent the main risk factors for atherosclerosis, are associated with an increased production of ROS by the endothelium [17]. Smoking and diabetes mellitus are involved also in the failure of DNA repair, and mitochondrial DNA is particularly susceptible to free radical damage [31, 32]. Indeed, mitochondrial dysfunctions can be caused by DNA damage and they are associated with atherosclerosis [33]. The increase of ROS derives also from loss of integrity of the mitochondrial respiratory chain, in particular at Complex I, which feeds back to increased DNA damage [34]. These changes are likely to affect all the cell types involved in atherosclerosis [35]. 

### 3.2. p66^Shc^, ROS, and Cardiovascular Diseases

Mitochondria are an essential ROS producer in heart and cardiovascular diseases. Several studies reveal the role of p66^Shc^ in ROS production within mitochondria and its involvement in CVDs [36, 37]. p66^Shc^ is also present in mitochondria-associated membranes (MAMs) and its levels change in an age-dependent manner [38, 39].

p66^Shc^ is a protein encoded by the ShcA gene [40, 41] that is expressed as three isoforms of about 46, 52, and 66 kDa in mammals. p66^Shc^ has an additional collagen homologous region (CH2) at its N-terminus [42]. 

**Figure 1:**
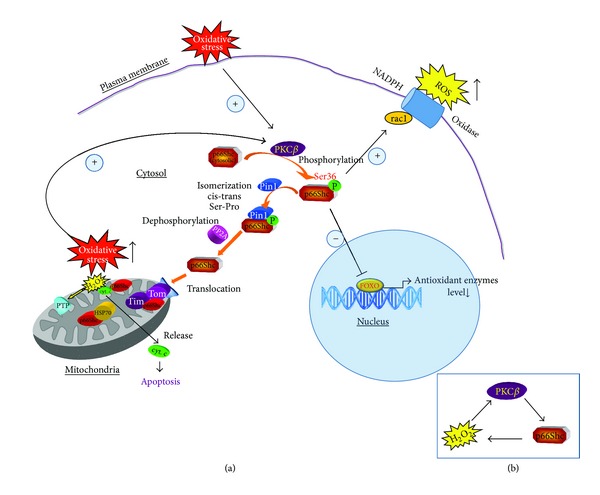
(a) Signal transduction pathway of p66^Shc^ in oxidative condition. Oxidative stress induces PKC*β* activation and p66^Shc^ phosphorylation allowing its recognition by Pin-1 and the transfer from the cytosol to the mitochondrion, where it induces PTP opening. In the nucleus, p66^Shc^  inhibits the FOXO transcription factors, causing a decrease of antioxidant enzymes level, while at the plasma membrane p66^Shc^ promotes ROS production by rac1 and NADPH oxidase activation. (b) Focusing on loop between PKC*β*,  p66^Shc^, and H_2_O_2_. PKC*β* activation by H_2_O_2_ promotes p66^Shc^ phosphorylation. Active p66^Shc^ induces H_2_O_2_ production, which in addition to H_2_O_2_ present endogenously leads to PKC*β* activation.

Some studies have shown that p66^Shc^ is very important for the regulation of the intracellular redox balance and oxidative stress levels. Many studies now support the fact that intracellular free radicals are reduced in cells lacking the p66^Shc^ gene. There are three mechanisms that involve p66^Shc^ in ROS formation. In the nucleus, p66^Shc^ inhibits the FOXO transcription factors, causing a decrease in the expression of ROS scavenging enzymes [43]. At the plasma membrane, p66^Shc^ promotes rac1 activation and triggers NADPH membrane oxidase ROS production. In addition, p66^Shc^ acts also in the mitochondrial intermembrane space (IMS). After serine phosphorylation by PKC*β* and prolyl-isomerization by Pin-1 [44], p66^Shc^ moves from the cytosol to the IMS, through the TIM/TOM mitochondrial import machinery. Here, a redox active region at its N-terminal mediates electron transfer from reduced cytochrome *c* to molecular oxygen and the production of H_2_O_2_ [45]. Oxidative stress activates PKC*β*, causing phosphorylation of p66^Shc^ and thus triggering its mitochondrial proapoptotic effects [46] (Figure 1(a)). It should be noted that after its translocation to mitochondria, p66^Shc^ induces mitochondrial H_2_O_2_ production and so further increases intracellular H_2_O_2_ levels; therefore, in this way it can maintain or increase PKC*β* activation in a kind of self-triggered control loop (Figure 1(b)) [46, 47].

The importance of p66^Shc^ in ROS signalling has suggested a role in aging and life span [48]; indeed, Migliaccio et al. demonstrated that its knockout increases life span in mice [49]. The same authors, however, have recently shown that this is observed only in mice living in protected laboratory conditions; when living in a natural environment, mice with a deletion of p66^Shc^ have a negative selective advantage [50].

The known role of p66^Shc^ in ROS generation is relevant to its involvement in CVDs. It has been demonstrated that p66Shc knockout (p66^Shc−/−^) mice are protected against vascular, cardiac, and renal impairment. On the contrary, overexpression of p66^Shc^ causes alteration of the mitochondrial network, leading to cytochrome *c* release and apoptosis. Napoli et al. demonstrated that mice with comparable lipid profiles, both in a low-fat condition as well as in a high-fat diet, had an increased early aortic lesion in p66^Shc^ wild-type strain, whereas p66^Shc−/−^ were protected. Of relevance, low predisposition to atherogenesis and reduced oxidative stress were coupled with reduced apoptosis in aortic lesions [51]. 

ROS generation is also one of the main pathophysiological mechanisms that links glucose metabolism to endothelial dysfunction and atherosclerosis. Hyperglycaemia plays also a central role in causing diabetic vascular complications. In particular, high glucose concentrations induce cellular events that increase the production of free radicals, which scavenge NO to form peroxynitrite (ONOO^−^). To demonstrate p66^Shc^ involvement, Menini et al. and Rota et al. carried out several studies on hyperglycaemia-induced ROS-mediated cardiovascular complications, and p66^Shc−/−^ mice were protected from cardiomyopathy [37]. Moreover, p66^Shc−/−^ diabetic mice showed an enhanced antioxidant defence and lower ROS generation [52]. Furthermore, p66^Shc^ is involved in endothelial dysfunction, vascular dysfunction and plaque formation [53], diabetes, myocardial remodelling atherosclerosis, and ischemia/reperfusion (I/R). It has been shown that vessels exhibit an increased production of ROS and, in turn, undergo functional impairment as a result of loss of NO bioavailability [54]. On the contrary, hearts from p66^Shc−/−^ mice display decreased ROS production and decreased myocardial injury caused by postischemic reperfusion [55]. Finally, a recent study by Noda et al. showed, in Japanese subjects, that p66^Shc^ gene expression levels in peripheral blood monocytes (PBMs) were significantly higher in coronary artery disease (CAD) patients, compared to non-CAD subjects [56].

### 3.3. Protein Kinase C, ROS, and Cardiovascular Diseases

Another class of proteins involved in CVDs is represented by specific isoforms of the protein kinase C (PKC) family. ROS trigger PKC through redox signalling: oxidation of critical cysteine residues on PKC isoforms is known to cause their activation and thus provides a mechanism by which ROS could turn on PKC.

Several works have identified critical roles for PKC family members in programming aspects of heart failure pathogenesis. Their activation can be cardioprotective and may mediate ischemic-preconditioning-(IPC-) induced protection [57]. Selective activation of PKC*ε* confers cardiac protection, whereas its selective inhibition abolishes protection induced by IPC [58]. During ischemic preconditioning intracellular ROS induce PCK*ε* activation and its translocation into mitochondria where it mediates several cardioprotective-signalling pathways and promotes cell survival [6]. In contrast, selective activation of PKC*δ* causes increased damage from ischemic insults both in neonatal cardiac myocytes and in adult isolated rat cardiac myocytes, whereas its inhibition results in protection [59]. The massive increase of intracellular ROS that occurs during I/R damage leads to PKC*δ* activation and leads its translocation to mitochondria and induction of cell death [6]. In addition to ROS activating PKC*δ*, the generation of ROS is in turn controlled by PKC*δ*. Knockout mice lacking PKC*δ* exhibit a loss of ROS formation by the endothelium when subjected to cell stress agents such as UV and TNF-*α* and are resistant to death induced by H_2_O_2_ [60].

PKCs are also involved in the activation of NADPH oxidase, a source of oxidative stress in vascular tissue of diabetes and insulin resistance state. Angiotensin II (ATII) has also been reported to induce O_2_^•−^ production, and both PKC and NADPH oxidase inhibitors are able to block this effect [61]. Experimental and clinical trials have shown that angiotensin 1-converting enzyme (ACE) inhibitors and ATII receptor blocker (ARB) have protective effects on diabetic nephropathy and cardiovascular events by the blocking of the renin-angiotensin system (RAS) [62, 63]. 

## 4. ROS in Adipocyte Differentiation and Obesity: Implication of p66^Shc^ and PKC

Obesity is a metabolic disease with pandemic proportions, against which no effective pharmacological treatments have been found so far. Obesity is defined as an excess accumulation of adipose tissue. During obesity, the excessive accumulation of lipids overstimulates the adipose tissue development by an increase in preadipocyte proliferation, differentiation into adipocyte, and size of mature adipocytes [64]. Obesity occurs in mammalian species when caloric intake exceeds energy expenditure. Cells experience stress as a result of “nutrient excess,” during which ROS production exceeds that required for normal physiological responses. 

### 4.1. ROS Roles in Obesity

It has been reported that obesity may induce systemic oxidative stress. Biomarkers of oxidative damage are higher in individuals with obesity and correlate directly with Body Mass Index (BMI) and the percentage of body fat [65]; in contrast, an inverse relationship between body fat, central adiposity, and antioxidant capacity has been suggested [66]. Several processes are involved in obesity-associated oxidative stress, caused by an overload of nutrients and in particular high-fat and high-carbohydrate meals. An increment of fat levels corresponds to increased energy storage, mitochondrial oxidation of nutrients, and oxidative stress, caused by an imbalance between ROS generation and ROS elimination by the cellular defence systems [67]. Oxidative stress derives from an increase of plasmatic concentration of free fatty acid (FFA) and increases leptin level, and leads also to inflammation, subnormal vascular reactivity, and insulin resistance [68]. Insulin resistance (IR) is a characteristic feature of type 2 diabetes and obesity and promotes atherogenesis in the absence of hyperglycemia [69]. Data by Du et al. show that IR increases mitochondrial ROS production, especially superoxide, from FFA by activation of proinflammatory signals implicated in hyperglycemia-induced vascular damage and inactivation of two enzymes involved in atherogenesis, prostacyclin synthase, and eNOS, leading to the development of atherosclerosis correlated to obesity and diabetes [12, 70].

Hyperglycaemic conditions and oxidative stress accelerate also the generation of advanced glycation end-products (AGEs), a complex group of compounds that derives from reaction between reducing sugars and amino residues present in proteins, lipids, and nucleic acids [71], mediating the complications of obesity, diabetes, and ischemic cardiovascular disease [72]. In CVDs, a mechanism proposed by several authors involves additional cross-linking on collagen by glycation of its free amino acids causing stiffness of blood vessels [73] or a reduction of LDL uptake by cell receptors because of their glycation on the apolipoprotein B and phospholipid components [74, 75]. Hyperglycemia also increases the glycation process, and glycation of proteolytic enzymes in diabetes reduces their efficiency [76, 77]. The ligand/receptor for advanced glycation end-products (RAGE) axis is also involved in several diseases related to obesity and atherothrombosis. The dysfunction of the adipose tissue seems to be associated with reduced sRAGE and adiponectin and increased oxidative stress, leading to platelet activation [78].

Both mitochondrial and endoplasmic reticulum (ER) stress responses can regulate or induce adaptation to the ROS production initiated by nutrient excess. Recently, *ob/ob* mice were reported to show upregulation of ER stress markers such as BiP, phosphorylated PERK, and phosphorylated *α*-subunit of eukaryotic translational initiating factor 2 (eIF2*α*) in adipose tissue and the liver [79]. Interestingly, several studies have demonstrated that FFA, which are elevated in obesity, have the potential to induce ER stress in various cells, including adipocytes [80]. However, the molecular mechanisms of obesity-induced ER stress in adipocytes are not fully understood yet. In a recent study, Kawasaki et al. showed that HFD-induced obesity causes ER stress and activates unfolded protein response (UPR) signalling in adipose tissue. Furthermore, the study found that alleviation of ER stress using chemical chaperones suppressed the inflammatory response that occurred in the adipose tissue of HFD-fed mice and improved insulin signalling. Therefore, this study revealed novel drug targets for obesity and opens the possibility that inhibition of ER stress may be an effective approach to reduce the risk of obesity and its complications [81].

In recent years, novel roles have been assigned to ROS, notably their involvement in the control of body weight by the central nervous system. Specifically, the location where ROS exert these newly described roles is the hypothalamus, where numerous neurons control our satiety, while others control our hunger behaviour. Such roles have been implicated as contributing factors underlying diverse findings such as the age-related decrease ability to lose weight and the caloric restriction-induced longevity [82].

A final important point to take into account is that epidemiological evidence clearly indicates that overnutrition at an organismal level also contributes to cancer development, so obesity is also associated with increased risk for several types of cancer [83, 84]. The molecular mechanisms underlying how obesity causes an increased risk of cancer are poorly understood. Understanding these molecular links may provide an avenue for preventive and therapeutic strategies to reduce cancer risk and mortality in an increasingly obese population.

### 4.2. Protein Kinase C, ROS, and Obesity

Numerous studies show that obesity may induce systemic oxidative stress and increase an ROS in adipocytes [13]. Excess glucose activates several biochemical mechanisms, including autoxidation of glyceraldehydes, glycation, methyl glyoxal and sorbitol production, hexosamine pathway, and oxidative phosphorylation, which cause an increase in ROS production [85]. High levels of glucose lead also to an increase in intracellular ROS that can promote PKC*β* activation [86]; once activated, PKC*β* induces p66^Shc^ phosphorylation, thus allowing p66^Shc^ to be recognized by Pin1, isomerized and imported into mitochondria, where p66^Shc^ acts as ROS producer and so further increases intracellular ROS levels (Figure 1(b)). Data by Nishikawa et al. show that the normalization of levels of ROS with an inhibitor of ETC complex II, an uncoupler of oxidative phosphorylation, the uncoupling protein-1, and the manganese superoxide dismutase leads to the prevention of glucose-induced activation of PKC isoforms [87]. 

Data throughout the literature indicate that an increase in ROS significantly affects white adipose tissue biology and leads to deregulated expression of inflammatory cytokines such as Tumor Necrosis Factor-*α* (TNF*α*) and insulin resistance, which could contribute to obesity-associated diabetes and CVDs [88]. Moreover, oxidative stress induced by ROS stimulates fat tissue development both in adipocyte culture systems and *in vivo*. Therefore, oxidative stress is induced by obesity, but at the same time it promotes fat accumulation. Lee et al. demonstrated that H_2_O_2_-induced oxidative stress facilitates the differentiation of preadipocytes into adipocytes by accelerating mitotic clonal expansion. This effect was explained through the positive regulation of major transcriptional activators such as CCAAT/Enhancer Binding Protein-*β* (C/EBP-*β*) and Peroxisomal Proliferator Activated Receptor-*γ* (PPAR-*γ*), which are able to coordinate the expression of genes involved in the adipocyte differentiation program [89]. Antioxidants such as flavonoids and N-acetylcysteine (NAC) inhibit both adipogenic transcription factors C/EBP-*β* and PPAR-*γ* expression, as well as adipogenic differentiation in 3T3-L1 preadipocytes [90, 91]. NAC was also shown to reduce ROS levels and fat accumulation in a concentration-dependent manner [91]. Moreover, animals on a high-fat diet (HFD) with the antioxidant NAC exhibited lower visceral fat and body weight [92]. Finally, ROS scavenging is associated with fat reduction in obese Zucker rats [93].

Aguiari et al. attributed an important role in adipogenic differentiation of mesenchymal stem cells, from both adipose tissue (adipose-derived stem cells (ADSc)) and muscle (muscle-derived stem cells (MDSCs)), to ROS and downstream effector kinases, in particular PKC*β* [86]. The serine/threonine-specific protein kinase PKC has been particularly implicated in the pathogenesis of obesity and insulin resistance [6, 94, 95]. Already in 1998, Fleming et al. [96] showed that PKC is an important player in adipocyte development. Then Bansode et al. demonstrated that overexpression of a dominant negative mutant of PKC*β*I blocked adipogenesis, suggesting that PKC*β*I is required in the induction of adipogenesis in 3T3-L1 preadipocytes and adipocytes. Subsequent studies demonstrated that mice lacking PKC*β* showed decreased fat in adipose tissue, liver, and muscle. These mice consumed 20–30% more food than wildtype, yet lost body weight, and the size of white fat depots was markedly decreased compared with that of wild-type litter-mates. The protection from obesity involves elevated oxygen consumption/energy expenditure and increased fatty acid oxidation in adipose tissue with concurrent increased mitochondrial biogenesis, upregulation of PGC-1*α* and UCP-2, and downregulation of perilipin [97]. Moreover, the same authors demonstrated that mice lacking PKC*β* are resistant to HFD-induced obesity, showing significantly reduced white adipose tissue (WAT) [98]. HFD selectively increased PKC*β* expression in obesity-prone C57BL/6J mice, specifically in WAT. Basal PKC*β* expression was also found to be elevated in WAT of obese *ob/ob *mice. Remarkably, PKC*β* ^−/−^ mice exhibited changes in lipid metabolism gene expression, and such alterations were accompanied by significant changes in serum adipokines [98].

**Figure 2:**
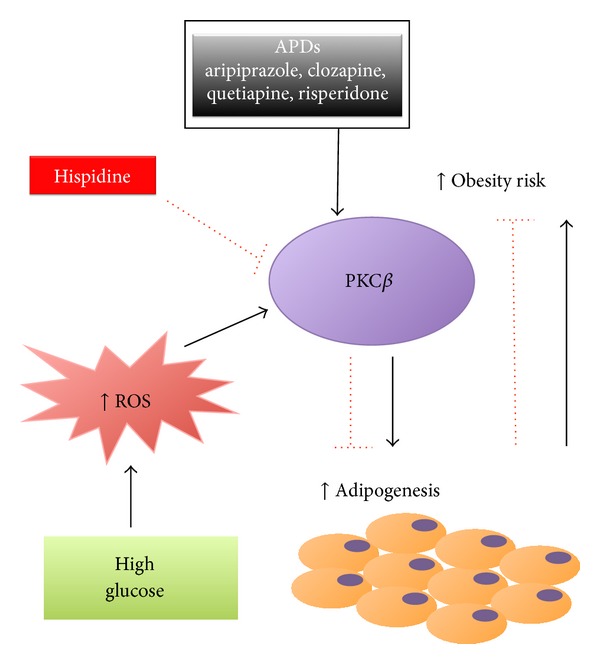
PKC*β* plays a key role in adipogenesis and obesity. Excess glucose increases ROS levels that lead to PKC*β* activation, and this activation is required in the induction of adipogenesis and consequently in the increasing of obesity risk. APDs influence adipogenic events by PKC*β* activation, and its inhibition through hispidine could prevent or delay the development of obesity.

These results raise the possibility that pharmacological manipulation of PKC*β* may lead to loss of body fat and may suggest novel therapeutic strategies for obesity and obesity-related disorders. In support of this notion, PKC*β* antagonists are currently undergoing clinical trials to reduce diabetes-linked complications [99]. Along similar lines, a new and interesting prospect has arisen recently. The results obtained by Pavan et al. indicate that atypical antipsychotics (APDs) influence adipogenic events through changes in the differentiation and proliferation of preadipocytes and MDSCs. These events are brought on by PKC*β* activation, as revealed both by the strong inhibitory effect of a specific PKC*β* inhibitor (hispidin) and through its genetic downregulation using siRNA [100]. This is strongly related to the well-known cellular response to high glucose which induces an increase in ROS production. These data identify a signalling route that could be a potential target for pharmacological approaches in the prevention of the well-known disadvantage of weight gain associated with APDs treatment, resulting frequently in severe obesity, dyslipidemia, and changes in insulin sensitivity, which are major risk factors associated with the development of cardiovascular complications [101]. Indeed, the authors hypothesize that the parallel administration of PKC*β* inhibitor, along with APDs, could prevent or delay the development of obesity and obesity-related disorders, introducing the hypothesis that the inhibition of PKC*β* could be therapeutically useful in conjunction with APDs (Figure 2). Further studies in this direction are needed to demonstrate *in vivo* that treatment with PKC*β* inhibitors protects from APD-induced weight gain and yet retain their ability to counteract anxiety.

As adiposity is related to oxidative stress and mitochondria are the main site of ROS generation, the role of mitochondria in white adipose tissue dysfunction during obesity could be a key event in obesity-induced oxidative stress and insulin resistance. A HFD has been shown to increase the ROS-emitting potential of mitochondria in both rats and humans, selectively in the adipose tissue [88]. 

### 4.3. p66^Shc^, ROS, and Obesity

ROS are also critical determinants of aging and age-associated diseases. PKC*β* acts as a signalling link between ROS and mitochondrial targets implicated in age-dependent organ deterioration. PKC*β*, activated by oxidative conditions in the cell, induces phosphorylation of p66^Shc^ and triggers mitochondrial accumulation of this protein [44]. Berniakovich et al. reported that p66^Shc−/−^ mice have decreased fat mass and resistance to diet-induced obesity and that p66^Shc^-generated ROS regulate insulin signalling through multiple mechanisms, including AKT phosphorylation, FOXO localization, and regulation of insulin target genes. Insulin, in fact, activates the redox enzyme-activity of p66^Shc^ in adipocytes, and H_2_O_2_ generated by p66^Shc^ reduces mitochondrial oxygen consumption and favours triglyceride accumulation through its effect on the insulin-signalling cascade. Mice without p66^Shc^ showed increased basal metabolism and insulin sensitivity of peripheral tissues and reduced fat development [7]. Moreover, in p66^Shc^ knockout animals, reduction of fat mass impairs their thermoregulation, as an evolutionary conserved adaption to cold [7, 102]. Furthermore, data by Ranieri et al. showed that effects of p66^Shc^ on mouse lifespan and on cardiovascular dysfunction [51] may be also ROS independent and a consequence of the role of p66^Shc^ in nutrient-related signalling. They investigated in fact interactions between p66^Shc^ and signalling cascades (mTOR/S6 kinase) triggered by insulin and nutrients in leptin-deficient Lep^Ob/Ob^ mice, a genetic model of obesity and IR. p66^Shc^ promotes the signal inhibitory phosphorylation of insulin receptor substrate 1 (IRS1) by connecting it with mTOR effector S6 kinase, demonstrating p66^Shc^ as a mediator of IR by excess nutrients [103]. Recent studies by Tomilov et al. made in p66^Shc−/−^ mice have confirmed the role of p66^Shc^ in insulin signalling; they have demonstrated that it is also the overexpression of fat of another isoform of Shc locus, p46, that is the likely cause of decreased adiposity and reduced insulin sensitivity [104].

## 5. Targeting ROS in Cardiovascular Diseases and Obesity: Therapeutic Potential

Antioxidants are agents that at low concentrations prevent or inhibit oxidation of oxidisable biomolecules, such as DNA, lipids, and proteins [105]. Superoxide dismutase (SOD), catalase, glutathione peroxidase, thioredoxin, and peroxiredoxin represent enzymatic antioxidants [106], while nonenzymatic antioxidants are vitamin E, vitamin C, and glutathione [107]. Other molecules, such as uric acid and bilirubin, are also antioxidants able to protect against CVDs [105]. In addition, there are two important carotenoids, *β*-carotene and lycopene, that are fat-soluble and can function as free radical scavengers to decrease initiation and propagation of fatty acid oxidation [108].

Antioxidants have been tested in several experimental and clinical models with mixed success. Lane et al. conducted a population-based study to examine the association between consumption of certain nutrients and prevalence of peripheral arterial disease (PAD), and they found that increased consumption of antioxidants, vitamin E and C was associated with reduced odds of PAD [109]. Other studies demonstrated the importance of vitamin E for protection against cardiac ischemia-reperfusion injury using vitamin E deficient animal models [110, 111]. These observations indicate that the modulation of oxidative stress by antioxidants appears to have a positive outcome in the prevention of CVDs. Despite this, the protective effects of vitamin E remain controversial, because it requires prolonged and very high levels of oral treatment to achieve cardiac concentrations that are protective from reperfusion injury [112, 113]. However, it should be considered that in these studies antioxidant agents might have been tested at inappropriate doses, or for inadequate durations, or that the wrong drug or combination of drugs has been used.

Therefore, regardless of these controversial data from clinical studies with no significant effects for the set up of appropriate treatments based on antioxidants, oxidative stress still remains a potential attractive target for CVDs prevention and therapy. Possible future therapies aimed at decreasing mitochondrial oxidative damage should also be considered.

In obesity, targeting adipocyte mitochondrial ROS production and increasing the overall antioxidant defence system are a challenge. A recent study suggested that antioxidant polyphenols (the major antioxidant micronutrients provided in the human diet by fruits, vegetables, and plant-derived beverages such as coffee and tea) can increase the antioxidant capacity of the body against obesity-induced oxidative stress through the prevention of mitochondrial alterations, while totally or partially protecting the cells against the consequences of oxidative stress [114].

Therefore inclusion of antioxidants in the diet may be indicated; indeed many foods, such as vegetables, fruits, red wine, and olive oil, contain phytonutrients that are soluble and can increase the antioxidant capacity [115, 116].

## 6. Conclusions

In this paper, we have focused on the involvement of oxidative stress in CVDs and obesity, in light of the fact that a strong correlation between these pathologies has been observed. Adipose tissue, particularly visceral fat, is in fact associated with the pathogenesis of diabetes, hypertension, and heart disease [117, 118].

ROS play an important role through highly regulated redox-sensitive signalling pathways, the adaptor protein p66^Shc^, and some isoforms of PKC family are relevant participants in this mechanism.

The use of antioxidants appears to be positive for the prevention of CVDs, while inhibitors of ER stress can represent novel drug targets for obesity. 

Understanding molecular links is fundamental to design new therapeutic strategies aimed at reducing the risk of developing these pathologies.
